# Less qualitative multiparametric magnetic resonance imaging in prostate cancer can underestimate extraprostatic extension in higher grade tumors

**DOI:** 10.1590/S1677-5538.IBJU.2023.0321

**Published:** 2024-03-18

**Authors:** Stephen Schmit, Sai Allu, Joshua Ray Tanzer, Rebecca Ortiz, Gyan Pareek, Elias Hyams

**Affiliations:** 1 Warren Alpert Medical School of Brown University The Minimally Invasive Urology Institute at The Miriam Hospital Division of Urology Providence RI The Minimally Invasive Urology Institute at The Miriam Hospital, Division of Urology, Warren Alpert Medical School of Brown University, Providence, RI

**Keywords:** Prostatic Neoplasms, Multiparametric Magnetic Resonance Imaging, Prostatectomy

## Abstract

**Background:**

Multiparametric magnetic resonance imaging (mpMRI) is increasingly used for risk stratification and preoperative staging of prostate cancer. It remains unclear how Grade Group (GG) interacts with the ability of mpMRI to determine the presence of extraprostatic extension (EPE) on surgical pathology.

**Methods:**

A retrospective review of a robotic assisted laparoscopic radical prostatectomy (RALP) database from 2016-2020 was performed. Radiology mpMRI reports by multiple attending radiologists and without clear standardization or quality control were retrospectively assessed for EPE findings and compared with surgical pathology reports. The data were stratified by biopsy-based GG and a multivariable cluster analysis was performed to incorporate additional preoperative variables (age at diagnosis, PSA, etc.). Hazard ratios were calculated to determine how mpMRI findings and radiographic EPE relate to positive surgical margins.

**Results:**

Two hundred and eighty nine patients underwent at least one mpMRI prior to RALP. Preoperative mpMRI demonstrated sensitivity of 39.3% and specificity of 88.8% for pathological EPE and had a negative predictive value (NPV) of 49.5%, and positive predictive value (PPV) of 84.0%. Stratification of NPV by GG yielded the following values: GG 1-5 (49.5%), GG 3-5 (40.8%), GG 4-5 (43.4%), and GG 5 (30.4%). Additionally, positive EPE on preoperative mpMRI was associated with a significantly decreased risk of positive surgical margins (RR: 0.655; 95% CI: 0.557-0.771).

**Conclusions:**

NPV of prostate mpMRI for EPE may be decreased for higher grade tumors. A detailed reference reading and image quality optimization may improve performance. However, urologists should exercise caution in nerve sparing approaches in these patients.

## INTRODUCTION

Multiparametric magnetic resonance imaging (mpMRI) is increasingly used for preoperative staging of prostate cancer and surgical planning for radical prostatectomy. Supplemental preoperative information from mpMRI may be especially important in surgical planning for high-risk cancers given the substantial heterogeneity in cancer-specific survival following radical prostatectomy (1). A 2019 meta-analysis found that preoperative mpMRI changed surgical planning in more than a third of cases overall and in 52% of high-risk tumors (2). In low-risk prostate cancer, mpMRI typically changes the surgical approach to wider resection through detection of more locally aggressive disease. In high-risk prostate cancer, however, preoperative mpMRI can lead to either wider resection (25%) or more aggressive nerve preservation (31%) as there may be reassurance from a lower risk scan (2).

Ongoing assessment of mpMRI performance is critical given its increasing use for surgical planning, which is supported by a growing body of evidence. The extent of prostate cancer at mpMRI may be independently associated with oncologic outcomes following prostatectomy, regardless of pathologic tumor stage (3). Radiographic features like estimate of extraprostatic extension (EPE), length of capsular contact (LCC), and seminal vesicle infiltration (SVI) are reported to be reliable predictors of prostate cancer in the histopathological T3 stage (4). However, urologists must consider the accuracy of mpMRI to avoid unnecessary removal of neurovascular bundles in lower risk patients, and inappropriate nerve preservation in higher-risk patients with EPE. While safe in appropriate patients, nerve-sparing approaches are independently associated with an increased risk of ipsilateral positive surgical margin (5). Preoperative mpMRI typically results in appropriate changes to the surgical plan in prostate cancer (6, 7). However, failure to perform a sufficiently wide dissection in a tumor with extraprostatic extension could have harmful clinical consequences. Thus, special attention should be given to the risk of a “false negative” preoperative mpMRI.

We hypothesized that with higher grade group (GG) detected by biopsy, mpMRI findings would have higher positive and lower negative predictive values for pathological EPE. Thus, our primary aim was to assess how preoperative GG and mpMRI findings interact to determine the presence of EPE on surgical pathology. A multivariable analysis was also pursued to identify specific populations associated with improved mpMRI performance in the detection of EPE. A secondary aim was to evaluate whether the presence of EPE on mpMRI was associated with positive surgical margins.

## MATERIAL AND METHODS

### Patients

A retrospective review of a robotic-assisted laparoscopic radical prostatectomy (RALP) database from a single academic institution from 2016-2020 was performed. All patients had localized prostate cancer confirmed by core biopsy. Patients from the registry were included in the sample if the following data were present: prostate core biopsy pathology, preoperative mpMRI imaging, and final surgical pathology. Imaging reports were retrospectively assessed for EPE findings to determine the sensitivity, specificity, positive predictive value (PPV), and negative predictive value (NPV) for pathological EPE. This study has been reviewed by a certified ethical board via an Institutional Review Board approval (IRB 1047794).

### Imaging Protocol

All mpMRI studies were completed with a 3T scanner without an endorectal coil and included T1, T2, diffusion weighted imaging (DWI), and dynamic contrast enhanced (DCE) sequences. Images were acquired before and after intravenous administration of Dotarem gadolinium-based contrast and kinetic analyses were performed using DynaCAD. The studies were interpreted by a group of attending radiologists from a single institution and assessed by PI-RADS v2.1. Attending radiologists were fellowship-trained in body imaging. The imaging reports were retrospectively reviewed to determine reader suspicion of EPE. The mpMRI report was considered positive for EPE if the radiologist explicitly expressed concern for EPE and identified lesion characteristics concerning for EPE, which included but were not limited to 1) broad contact of the tumor with the prostatic capsule >1 cm and concerning for EPE, 2) irregularity or bulging of the prostatic capsule concerning for EPE, or 3) gross visualization of EPE.

### Statistical Analysis

To assess the primary aim, patients were assigned GG based on Gleason scores from preoperative core biopsy, as outlined by the International Society of Urological Pathology histological definitions (8). Data was stratified by predicted GG to determine the relationship between tumor grade and the accuracy of mpMRI findings. Sensitivity, specificity, PPV, and NPV were calculated for each subgroup. Cluster analysis methodology was used for multivariable analysis to group patients based on similarity of multiple preoperative variables including age at diagnosis, family history of prostate cancer, body mass index (BMI), prostate volume estimated by mpMRI, prostate specific antigen (PSA), number of positive biopsy cores, Gleason score, and preoperative grade group based on biopsy. Clustering empirically groups patients based on the numeric similarity of the data provided, this approach identifies clinically meaningful groups of patients based on their disease presentation and thus maintains fidelity between patients and their multiple preoperative traits. The Kamila algorithm was used for identifying clusters (9). After clusters were identified, sensitivity, specificity, PPV, and NPV were estimated and compared by generalized linear mixed effects modeling (10).

To assess the secondary aim, the hazard ratio was calculated to determine how concern for EPE relates to positive surgical margins. The Python programming language was used for data processing (i.e. grouping patients by GG) and Microsoft Excel was used to generate performance parameters (sensitivity, specificity, PPV and NPV) and hazard ratios. The clustering analysis was completed in the R programming language.

## RESULTS

**Figure 1:**
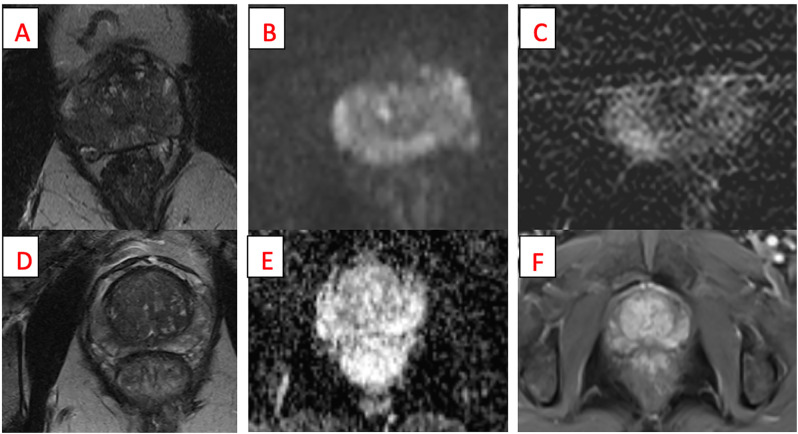
Example mpMRI images yielding false negative and false positive findings for extraprostatic extension (EPE). Top row: Axial T2 weighted image (panel A), diffusion weighted imaging (DWI) (panel B), and dynamic contrast enhanced (DCE) image (panel C) of false positive EPE finding. On pathology, grade group (GG) 4 prostate cancer was identified at the right mid to apex peripheral zone close to the capsular margin, but no EPE was identified. Bottom row: Axial T2 weighted image (panel D), DWI (panel E), and DCE image (panel F) of false negative EPE finding. On pathology, GG 5 prostate cancer was identified at the right mid to base peripheral zone and non-focal EPE was identified.

A total of 289 patients underwent at least one mpMRI prior to RALP for localized prostate cancer. The average patient age at diagnosis was 61.5 ± 5.9 years. The overall performance of mpMRI for pathological EPE demonstrated a sensitivity of 39.3%, specificity of 88.8%, negative predictive value (NPV) of 49.5%, and positive predictive value (PPV) of 84.0%. Figure-1 demonstrates the radiographic appearance of false negative and false positive mpMRI findings within this sample. Subgroup analysis revealed marginal improvement in sensitivity for higher grade tumors, with a sensitivity of 54.3% for GG 5 tumors. NPV decreased for higher grade tumors, with an NPV of 30.4% for GG 5 tumors. See Table-1 for full subgroup analysis. Concern for EPE was more prevalent for higher grade tumors stratified by GG when GG was determined by final pathology (Table-2). A multivariable clustering analysis was also performed to identify groups with significantly different accuracy parameters in the detection of EPE. Four distinct clusters were identified (Table-3). Cluster 2 was excluded from further analyses due to small sample size (n=4). Cluster 4 had the most average positive biopsy cores (8.64, 95% CI 8.09-9.23), significantly more than clusters 1 and 3. Cluster 4 was significantly more accurate than cluster 3 for sensitivity (*p=0.0123*), but significantly less accurate for specificity (*p=0.0177*) and NPV (*p=0.0046*). Cluster 3 was also the least likely to have a positive finding of EPE on MRI (compared to cluster 1 *p=0.0570*, compared to cluster *4 p=0.0012*).

**Table 1:** mpMRI detection of extraprostatic extension (EPE) by increasing tumor grade.

Biopsy Grade Group	n	EPE Concern on MRI	EPE Prevalence on Pathology	Sensitivity	Specificity	Negative Predictive Value	Positive Predictive Value
**GG 1-5**	289/289 (100%)	81/289 (28.0%)	173/289 (59.9%)	39.3%	88.8%	49.5%	84.0%
**GG 3-5**	176/289 (60.9%)	56/176 (31.8%)	121/176 (68.8%)	41.3%	89.1%	40.8%	89.3%
**GG 4-5**	116/289 (40.1%)	40/116 (34.5%)	80/116 (69.0%)	46.3%	91.7%	43.4%	92.5%
**GG 5**	43/289 (14.9%)	20/43 (46.5%)	35/43 (81.2%)	54.3%	87.5%	30.4%	95.0%

**Table 2:** Cross table comparing extraprostatic extension prevalence after stratification by biopsy-determined grade group and final pathology-determined grade group.

	Grade Group	n	EPE Concern on mpMRI	EPE Prevalence on Pathology
Biopsy Grade Group	GG 1-5	289/289 (100%)	81/289 (59.9%)	173/289 (59.9%)
	GG 3-5	176/289 (60.9%)	56/176 (31.8%)	121/176 (68.8%)
	GG 4-5	116/289 (40.1%)	40/116 (34.5%)	80/116 (69.0%)
	GG 5	43/289 (14.9%)	20/43 (46.5%)	35/43 (81.2%)
Final Pathology Grade Group	GG 1-5	289/289 (100%)	81/289 (59.9%)	173/289 (59.9%)
	GG 3-5	141/289 (48.8%)	48/141 (34.0%)	107/141 (75.9%)
	GG 4-5	68/289 (23.5%)	32/68 (47.1%)	60/68 (88.2%)
	GG 5	49/289 (17.0%)	25/49 (51.0%)	45/49 (91.8%)

**Table 3:** Clustering analysis of preoperative mpMRI accuracy in the detection of extraprostatic extention (EPE).

Trait	Cluster 1	Cluster 2	Cluster 3	Cluster 4
**Demographics**				
Age at Diagnosis	62 [60-63]	57 [54-60]	61 [60-62]	62 [61-63]
Family History of Prostate Cancer	100% [100%-100%]	0% [0%-0%]	0% [0%-0%]	15% [9%-23%]
BMI	28 [27-29]	35 [29-42]	28 [27-29]	28 [27-29]
**Disease State**				
Preoperative Prostate Volume	44 [36-55]	48 [20-231]	53 [44-65]	41 [34-51]
PSA	8 [7-10]	7 [3-35]	8 [6-9]	10 [8-13]
Number of Positive Cores	5.04 [4.59-5.54]	7.00 [4.56-10.74]	3.43 [3.09-3.81]	8.64 [8.09-9.23]
Gleason Score	7.74 [7.55-7.93]	8.00 [6.87-9.13]	7.28 [7.13-7.43]	7.45 [7.29-7.6]
Grade Group	2.49 [2.37-2.6]	2.67 [2.01-3.32]	2.22 [2.09-2.34]	2.29 [2.18-2.4]
Group Frequencies	86	3	102	103
**Predictive Value of MRI and Pathology Reports**				
MRI % Positive	23% [14%-33%]	-	16% [10%-25%]	37% [27%-47%]
Pathologist % positive	65% [53%-75%]	-	48% [38%-59%]	71% [61%-79%]
Sensitivity	38% [26%-51%]	-	27% [17%-41%]	49% [39%-60%]
Specificity	91% [80%-96%]	-	93% [86%-97%]	79% [65%-88%]
Positive predictive value	87% [73%-94%]	-	75% [56%-88%]	86% [76%-92%]
Negative predictive value	48% [36%-59%]	-	63% [53%-72%]	37% [26%-49%]

A secondary aim of this study was to assess the relationship between mpMRI findings and positive surgical margins. A total of 294 patients had available reports on margin status. Positive EPE on preoperative mpMRI was associated with a significantly decreased risk of positive surgical margins (RR: 0.655; 95% CI: 0.557-0.771). Table-4 represents the distribution of margin status by mpMRI findings for EPE.

**Table 4:** “Confusion matrix” demonstrating relationship of mpMRI findings (positive vs. negative for EPE [extraprostatic extension]) to surgical margin status on final pathology (positive vs. negative margin. EPE+ on mpMRI was associated with a lower risk of positive surgical margin (RR: 0.655, 95% CI 0.557-0.771).

		EPE status on mpMRI	
		+EPE	-EPE
Surgical margin status	-margin	168	56
	+margins	44	26

## DISCUSSION

Imaging of the prostate with mpMRI has become increasingly valuable in prostate cancer management as image quality has improved with advancements like high-field strength magnets. For initial management, mpMRI is particularly useful for improving detection of clinically significant prostate cancer and guiding both transrectal and transperineal targeted biopsies (11-13). Although mpMRI is not yet integrated in guidelines for preoperative planning, mpMRI findings are likely to influence the surgical plan especially in high-risk tumors (14). Furthermore, mpMRI findings have direct clinical implications and may even predict positive surgical margins or be used to guide intraoperative frozen-section analysis (15, 16). Our study evaluated the accuracy of preoperative mpMRI by isolating tumor grade. We determined that the sensitivity of mpMRI for EPE improved with higher grade prostate tumors but remained low even with GG 5 tumors. NPV for EPE was low and decreased further with higher grade tumors. The low NPV suggests a large proportion of falsely reassuring mpMRI findings. This study confirms that a higher-grade group is associated with low NPV for high risk tumors, similar to a prior study using D’Amico risk criteria published in 2013. Somford et al. studied 48 high risk patients (corresponding to predicted GG 4-5 prostate cancer) from a sample of 187 and found an NPV of 38.1% (17). We found a similar NPV of 43.4% from a larger sample of 117 predicted GG 4-5 patients. Our study also evaluated GG 5 patients individually and confirmed an even lower NPV of 30.4% for this group. The lower NPV in this study may reflect an increasing trend towards surgical treatment of higher stage disease. Urologists should exercise caution in the setting of high-grade tumors when evaluating a preoperative mpMRI and considering an intra-fascial nerve-sparing procedure. In this setting, GG may serve as a convenient marker for expected mpMRI performance.

The clustering analysis allowed for the incorporation of multiple preoperative variables (age at diagnosis, PSA, number of positive biopsy cores, etc.) to explore how these factors interact with mpMRI accuracy. This analysis has clinical utility for identifying categories of patients that can expect differing mpMRI performance in the evaluation of their disease. Clustering empirically identified 4 groups with varying mpMPRI performance in EPE detection. Cluster 4 was notable for including patients with significantly more positive biopsy cores than the other clusters. Cluster 4 also demonstrated the lowest NPV of any cluster, and this difference reached statistical significance when compared to cluster 3. Cluster 4 had no significant differences in PSA, Gleason score, or other preoperative variables when compared to the other clusters (Table-3). This analysis suggests the importance of caution in interpreting a negative mpMRI finding in the setting of higher volume disease. Cluster 4 demonstrates that higher volume disease may be an independent risk factor for impaired NPV of mpMRI for EPE, perhaps due to the higher prevalence of EPE in these patients.

Paradoxically, positive EPE on preoperative mpMRI was associated with a significantly lower risk of positive surgical margins following RALP. This finding stands in contrast to previous studies and may be counterintuitive (5). A possible explanation for this association with decreased positive margins is the practice of performing wider dissections when there is radiographic concern for EPE. This effect may be appropriate due to the relatively high specificity and positive predictive value of mpMRI for pathological EPE.

The meta-analysis by Kozikowski et al. demonstrated that when preoperative mpMRI did not change the surgical plan, the unchanged decision was more often appropriate than when mpMRI did change the surgical plan (2). The present study reveals the mixed performance of mpMRI for detecting EPE in high grade tumors and in the setting of higher volume disease. Thus, we argue that for tumors with these high-risk characteristics, the risk of EPE is high and may not always be evident radiographically. Mehralivand et al. demonstrated that formal grading systems to determine EPE risk based on factors like capsular irregularities and curvilinear tumor-capsule contact length may improve mpMRI accuracy (18). Combining clinical parameters, such as prostate-specific antigen (PSA) and Gleason score, with mpMRI findings also improved pathologic EPE prediction (18). Our clustering analysis supports this multifaceted approach by demonstrating the presence of 4 groups with significantly different mpMRI performance parameters. Better understanding of mpMRI accuracy is needed and will help guide the smooth integration of mpMRI data into risk stratification systems like that developed by Boschheidgen et al., which provides a more holistic representation of aggressiveness (19). Advancements in artificial intelligence may modify this landscape further as new models are emerging that can aid in EPE detection by correlating radiomics features from mpMRI with EPE risk (20). An important additional consideration might be the differentiation of microscopic EPE compared to clear EPE, which would not be evident on mpMRI. However, clinical implications of these findings may be different. Formal mpMRI grading systems, multimodal risk stratification systems, and/or further technical improvement plus possible artificial intelligence integration may improve mpMRI to better predict the presence of EPE especially in higher grade tumors (18-20).

## LIMITATIONS

One important limitation of our study was the inability to account for mpMRI imaging quality. As demonstrated by Figure-1, imaging quality may have varied substantially in our study. Future studies that utilize imaging reports assessed by the prostate imaging quality (PI-QUAL) scoring system will be useful to determine how EPE detection interacts with imaging quality (21). We were also limited by variation in reader experience given that the images were interpreted by multiple radiologists at a single institution. The accuracy of mpMRI for EPE detection improves with reader experience (22). Additional mpMRI information including apparent diffusion coefficient (ADC) values, lesion diameters, tumor volume, and length of capsular contact were not consistently reported in the dataset. Multivariable analyses that include these parameters may further clarify the accuracy of mpMRI in EPE detection. Formal EPE scoring with the 3-point rubric developed by Mehralivand et al. was not included in this study but similarly may have improved mpMRI performance (18). Our study was also limited by its retrospective nature; prospective studies will be helpful to clarify how mpMRI and other disease characteristics can interact to predict EPE and thereby inform the surgical approach.

## CONCLUSIONS

Preoperative mpMRI provides important insight for surgical planning in the setting of prostate cancer but should be cautiously used as justification for nerve-sparing in high grade disease and in patients with high volume disease given low NPV for EPE. However, radiologist experience was not assessed in this study and is likely a strong contributor to performance metrics. The increasing trend of surgical management for high-risk tumors warrants continued assessment of mpMRI performance. Future studies are needed to assess how mpMRI performance interacts with image quality (i.e. PI-QUAL scores) and radiographic lesion characteristics (i.e. PI-RADS scores, ADC, and lesion diameter).

## Abbreviations

mpMRI =: multiparametric magnetic resonance imaging
EPE =: extraprostatic extension
GG =: grade group
RALP =: robotic assisted laparoscopic radical prostatectomy
NPV =: negative predictive value
PPV =: positive predictive value
BMI =: body mass index
PSA =: prostate specific antigen
